# Enhancing reader confidence through dual-time imaging and alternative reconstruction algorithms with [^18^F]PSMA-1007 PET/CT imaging in local relapsed castration-sensitive prostate cancer?

**DOI:** 10.1186/s13550-026-01407-x

**Published:** 2026-03-03

**Authors:** Emil Novruzov, Gabriel Sheikh, Anna Feisthauer, Dominik Schmitt, Katalin Mattes-György, Christina Antke, Julian Kuhlmann, Yuriko Mori, Jan Henke, Jens Cardinale, Matthias Boschheidgen, Jan Philipp Radtke, Günter Niegisch, Peter Albers, Gerald Antoch, Rudolf A. Werner, Frederik L. Giesel, Eduards Mamlins

**Affiliations:** 1 Department of Nuclear Medicine, Medical Faculty, University Hospital Duesseldorf, Heinrich Heine University Duesseldorf, Moorenstrasse 5, 40225 Duesseldorf, Germany; 2 Department of Nuclear Medicine, LMU University Hospital, LMU Munich, Munich, Germany; 3 Department of Diagnostic and Interventional Radiology, Medical Faculty, University Hospital Duesseldorf, Heinrich Heine University Duesseldorf, 40225 Duesseldorf, Germany; 4 Department of Urology, Medical Faculty, University Hospital Duesseldorf, Heinrich Heine University Duesseldorf, 40225 Duesseldorf, Germany; 5 Centre for Integrated Oncology (CIO) Düsseldorf, CIO Aachen-Bonn-Cologne- Duesseldorf, 40225 Duesseldorf, Germany

**Keywords:** TOF and PSF reconstruction algorithm, PSMA-RADS framework, [^18^F]PSMA-1007 imaging, Delayed imaging, Biochemical recurrence, Prostate cancer

## Abstract

**Background:**

The current national and international guidelines recommend the use of PSMA imaging in proven BCR including very low levels of PSA (< 0.5 ng/ml), which is associated with a certain rate of false-negative results in regular clinical care. To address this issue, research efforts have focused on improving acquisition protocols via the implementation of delayed images or alternative reconstruction algorithms. One of the mainstays of this approach is the use of standardized reporting systems in regular clinical care and research setting to enhance reliability and reproducibility compared with unstructured confidence assessment. Thus, we aimed to investigate the added benefit of delayed imaging and the utility of reconstruction algorithms for the discrimination of equivocal [^18^F]PSMA-1007 findings in prostate bed.

**Results:**

This monocentric, retrospective study enrolled 36 biologically male patients who underwent dual-time contrast-enhanced [^18^F]PSMA-1007 PET/CT scan between October 2021 and February 2024 due to BCR at a tertiary referral hospital. Histopathology after salvage surgery was considered as the gold standard, while clinical, biochemical, and radiological follow-up served as composite reference standards after reviewing the follow-up information. The minimum follow-up per patient was 15 months. The retrospective reading of the early-phase images revealed equivocal PSMA findings, i.e. PSMA-RADS 3 A, in the prostate bed in 12/36 patients (33%), with a total median SUV_max_ of 5.1 (3.7–6.7). The favourable results with delayed [^18^F]PSMA-1007 imaging led to upgrading of reporting in 8 out of 12 patients (75%) from PSMA-RADS 3 A to PSMA-RADS 4/5.

**Conclusion:**

In conclusion, based upon the study results, we suggest the introduction of a new algorithm to enhance and streamline the imaging decision-process, which would prevent avoidable follow-up scans or further imaging modalities and spare economic resources. Yet, further large-scale studies are warranted to validate the additive effect of this algorithm in regular clinical care.

**Supplementary Information:**

The online version contains supplementary material available at 10.1186/s13550-026-01407-x.

## Introduction

The routine use of PSMA ligands is meanwhile an indispensable part of regular clinical care in prostate cancer management. ^68^Ga-labelled PSMA ligands are still most commonly used but have limitations such as a short half-life, limited batch-size, inadequacy for large-scale production and restricted spatial image resolution. These drawbacks are overcome by the development and introduction of ^18^F-labelled PSMA ligands such as [^18^F]PSMA-1007, as the current literature data indicates no substantial difference between in diagnostic performance of the three most commonly used PSMA-radiotracers ([^68^Ga]PSMA-11, [^18^F]PSMA-1007, [^18^F]DCFPyl). Recently, [^18^F]PSMA-1007 gained regulatory approval after successful completion of the clinical phase III study (NCT04102553) in western Europe [1–6]. The unique feature of [^18^F]PSMA-1007 is its predominant hepatobiliary excretion pathway sparing overspill effects of radiotracer from the urinary bladder to the prostate bed or the suspected lymph nodes in the vicinity of urinary bladder. This feature offers advantages particularly in the detection of local relapse in the setting of biochemical recurrence (BCR) of prostate cancer [7, 8].

The current national and international guidelines suggest the use of PSMA imaging in proven BCR including very low levels of PSA (< 0.5 ng/ml), which is associated with a certain rate of false-negative results in regular clinical care. For instance, Mingels et al. reported an overall rate of 91% positive findings in [^18^F]PSMA-1007 imaging with a sensitivity and specifity of 95% (CI: 0.90–0.98) and 89% (CI: 0.83–0.93), respectively [9–12]. To address this issue, research efforts have focused on improving structured reporting of findings to reduce reader dependent false-negativity and the use of more sophisticated acquisition protocols via the implementation of delayed images or alternative reconstruction algorithms [13–15].

Delayed imaging has been mostly investigated in combination with intravenous furosemide use on ^68^Ga-labelled PSMA ligands due to its renal clearance [16–18]. Owing to the different pharmacokinetics of [^18^F]PSMA-1007 imaging, these protocols are in most parts not applicable for [^18^F]PSMA-1007 imaging. Rahbar et al. investigated, to our best knowledge as the only research group, the dual-time [^18^F]PSMA-1007 PET acquisition with comparison of early and late acquisition times of 60 and 120 min after tracer administration. The authors reported a 70% increase in the median SUV_max_ in delayed images so that they proposed a standard acquisition time of 120 min to meet the best trade-off for an increased lesion detection rate [19]. According to the current approval instructions of [^18^F]PSMA-1007, image acquisition must occur between 90 and 120 min after tracer administration which necessitates refinement of dual-time imaging with [^18^F]PSMA-1007 [20].

Iterative reconstruction algorithms, i.e. ordered subset expectation maximization (OSEM), represent the current standards for PET image reconstruction which ensures finding the trade-off between improving signal-to-noise ratio (SNR) and reducing partial volume effect (PVE). In particular, correct quantification of tracer uptake in very small lesions of < 10 mm underlie certain drawbacks of the calculation of the standard uptake value (SUV), as this is substantially affected by various factors like duration of the uptake period, reconstruction methods and PVE. Recently developed PET/CT scanners have been equipped with the combination of time-of-flight (TOF) and point spread function (PSF) that improve SNR and compensate spatial variances. Reconstruction algorithms that combine TOF and PSF lead to increase of SUV_max_ value by > 30% in clinical studies and, therefore, OSEM reconstruction algorithms are considered to be more suitable for reporting consistency [21–25]. Khatri et al. investigated the added value of the combination of PSF and TOF in a cohort of 30 patients using [^18^F]DCFPyL and underscored the potential of this reconstruction algorithm in the differentiation of equivocal PSMA-avid findings [14]. To our best knowledge, there is no data concerning the specific role of this reconstruction algorithm for [^18^F]PSMA-1007 in the literature.

The third pillar for the optimization of PSMA-targeted imaging is the use of, standardized reporting systems in regular clinical care and research setting, as this would enhance reliability and reproducibility compared with unstructured confidence assessment. Essentially, guidelines recommend the use of a 5-point scale with an increasing likelihood of malignancy on the basis of the confidence of reader for PSMA-targeted imaging [13]. Thus, the recently introduced PSMA-RADS version 2.0 seems to be an optimized reporting framework meeting those prerequisites. This framework foresees the assignment of uncertain PSMA findings as PSMA-RADS 3 A, which would depict a suspected local relapse in the prostate bed [26, 27].

Our study aimed to investigate the added benefit of delayed imaging and the utility of reconstruction algorithm with combination of TOF and PSF for the discrimination of equivocal [^18^F]PSMA-1007 findings in the prostate bed in cases of suspected local relapse.

## Materials & methods

### Patient cohort

This monocentric, retrospective study enrolled 36 biologically male patients who underwent dual-time contrast-enhanced [^18^F]PSMA-1007 PET/CT scan between October 2021 and February 2024 due to BCR at a tertiary referral hospital. Thirty-four patients had undergone curative-intent radical prostatectomy, while two patients had curative-intent radiotherapy. An increase in PSA in two consecutive follow-up controls was considered as BCR in accordance with current guidelines [28, 29]. Our institutional protocol foresees the contrast enhanced acquisition of [^18^F]PSMA-1007 PET/CT scans with sporadic acquisition of delayed images with low-dose CT in cases of equivocal PSMA findings.

Imaging data were retrieved from in-house picture archiving and communication system (PACS) and electronic patient records were additionally selectively analyzed to gather further information on medical history. The data were pseudonymized and retrospectively analyzed. The study received approval from the Ethical Committee of the Medical Faculty of Heinrich Heine University Duesseldorf, Germany (Study-Nr.: 2024–2735).

### PET/CT acquisition

Following the intravenous application of [^18^F]PSMA-1007 (injected mean activity of 250 MBq (± 17)), the acquisition of whole-body, contrast-enhanced PET/CT imaging was performed after a mean of 90 (± 5) minutes, while delayed imaging of the pelvis was conducted after 178 min (± 10) with low-dose CT for anatomic localization and attenuation correction. All PET/CT scans were acquired in 3D mode with a body weight-adjusted acquisition time of 3–5 min/bed position with a Siemens Biograph 128 mCT PET/CT scanner (Siemens, Erlangen, Germany) in accordance with our institutional protocol (Supplementary Table S1).

Image acquisition (early images) was performed in the supine position from the skull base to the mid-thigh as a whole-body PET/CT scan. The CT component was performed 70 s after intravenous injection of a weight-adapted dose of iodinated contrast agent with a maximal dose of 80 ml (Accupaque 300, GE Healthcare, Munich, Germany) followed by a 60 ml bolus of physiological saline. The subsequent PET scan was acquired in the caudocranial direction in accordance with guidelines [10]. The delayed PET/CT scan of the pelvis region was acquired as mentioned above 178 (± 10) minutes after radiotracer injection following urinary bladder emptying. No additional iodinated CT contrast agent was injected for delayed PET/CT imaging. All patients were monitored for any adverse effects up to 30 min after the end of the examination.

### Image analysis

Both early and delayed [^18^F]PSMA-1007 images alongside the medical history of patients were visualized and analyzed by two experienced, board-certified nuclear medicine physicians (first and last authors, respectively) and unclear findings were resolved by consensus. The [^18^F]PSMA-1007 uptake in the prostate bed was evaluated using SUV metrics and qualitative assessment employing OSEM as well as of combined TOF and PSF reconstructions, in accordance with PSMA-RADS V2 for both early and delayed images.

Tracer uptake in lesions was quantified by the mean and maximum standardized uptake values (SUV_mean_ and SUV_max_). The tumor-to-background ratio (TBR) was derived by dividing the SUV_max_ of the tumor lesions by the SUV_mean_ of the skeletal muscle in the pelvis region (Obturator internus muscle) and the blood pool in the right common iliac artery. Circular regions of interest (ROIs) were placed over the suspected lesions and normal organs (muscle, vessel) in transaxial slices on early- and delayed scans via the dedicated reading software program Hermes Medical Imaging (Suite v6.1, Hermes Medical Solutions AB, Strandbergsgatan 16, 11251 Stockholm, Sweden). These were then automatically adapted to a three-dimensional volume of interest (VOI) at 40% isocontour. Previous experience in molecular imaging regarded a variability of SUV measurements of up to 10% as normal in iterative OSEM reconstructed PET imaging [30]. Histopathology after salvage surgery is considered as the gold standard, while clinical, biochemical, and radiological follow-up served as composite reference standards after reviewing the follow-up information. Follow-up time was defined as the time interval between the index PET/CT scan and the last documented clinical, biochemical or imaging assessment available in the electronic medical record. The minimum follow-up per patient was 15 months.

### Statistical analysis

We used descriptive analyses for demographics, tumor characteristics, and tracer uptake. SUV-metrics in tumor and normal tissues as well as TBRs were analyzed via paired t tests or Wilcoxon signed rank tests. A p value of < 0.05 was considered to indicate statistical significance. All the statistical analyses or graphical illustrations were performed using Excel Version 2311 (Microsoft^®^ Excel^®^ 2021 MSO), DataTab statistics tool, SigmaStat Version 3.5 (Systat Software, Inc., San Jose, CA, USA) and SigmaPlot Version 11.0 (Systat Software, Inc., San Jose, CA, USA) [31]. In this study, data cleansing was performed to identify and correct any errors, inconsistencies, or missing values in the dataset, thus improving the overall quality and reliability of the data.

**Table 1:** Baseline patient characteristics

Parameter	Value
Age (mean ± SD) in years	71 (± 5)
ISUP (Prior Treatment)	
ISUP 1	2
ISUP 2	10
ISUP 3	9
ISUP 4	4
ISUP 5	6
Robot assisted Radical Prostatectomy	34
Curative-intent radiotherapy	2
PSA nadir (median, range) in ng/ml	0.04 (0.02–1.85)
PSA at the time of PET/CT scan (median, range) in ng/ml	0.58 (0.2–4.8)
Biochemical recurrence free time (between therapy and PET/CT scan) (median, range) in years	3.4 (0.3–19.4)
Injected [^18^F]PSMA-1007 Activity (mean ± SD) in MBq	250 (± 17)
Acquisition time for early images (after IV tracer injection) (mean ± SD) in minutes	90 (± 5)
Acquisition time for late images (after IV tracer injection) (mean ± SD) in minutes	178 (± 10)

*ISUP: International Society of Urological Pathology

*PSA: Prostate specific antigen

## Results

### Patient demographics and imaging parameters

**Fig. 1:**
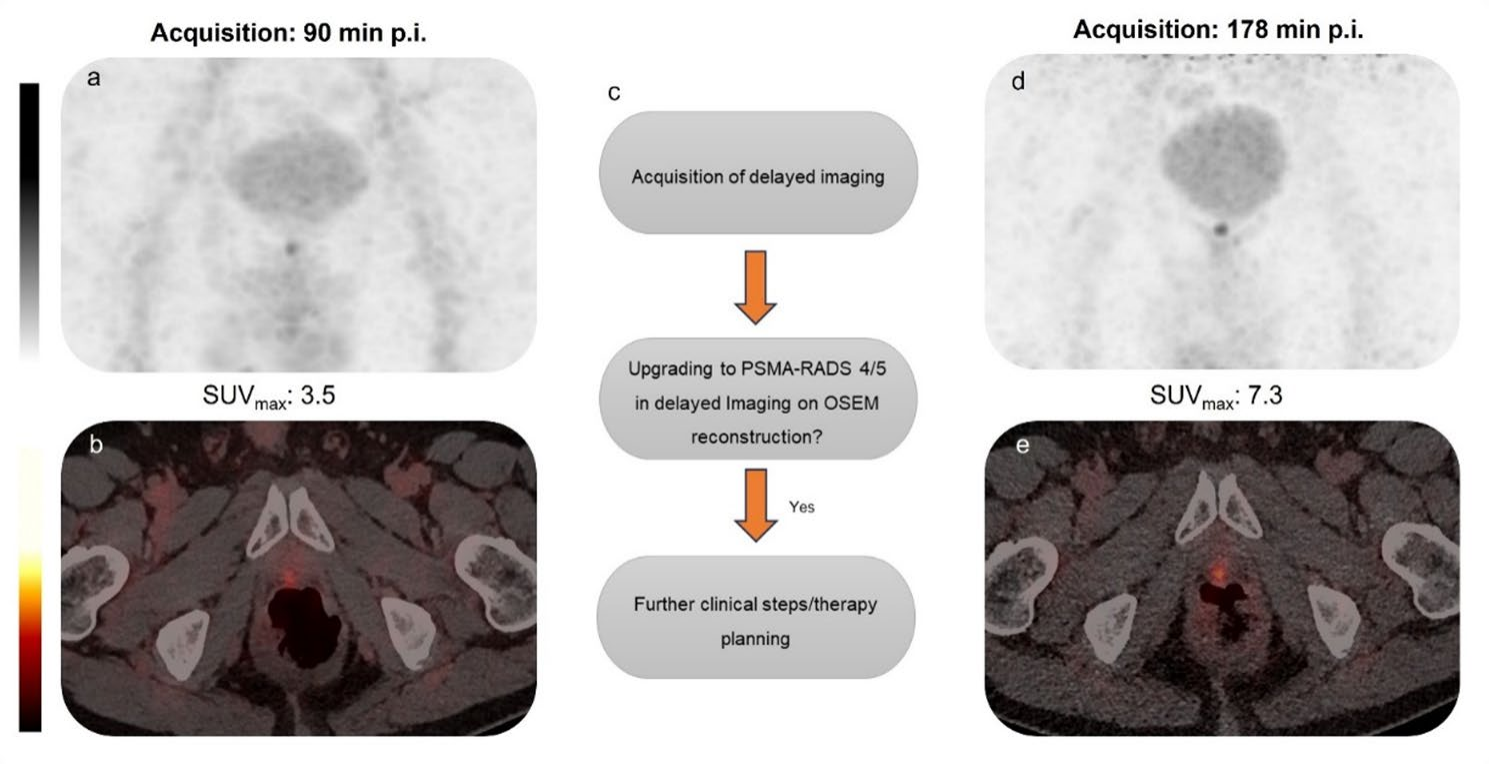
Depiction of a clinically challenging case of an equivocal tracer uptake in the prostate bed with no anatomical correlate in a 62-years-old local-relapse suspected patient (**a** & **b** in early-phase images vs. **c** & **d** in delayed-phase images)

In total, 36 patients underwent recurrence work-up at a median PSA level of 0.58 (0.2–4.8) ng/ml. The baseline patient characteristics are outlined in Table 1. A standard early-phase, whole-body [^18^F]PSMA-1007 PET/CT scan was performed with contrast enhancement 90 (± 5) minutes after the administration of a mean activity of 250 MBq (± 17). The retrospective reading of the early-phase images revealed equivocal PSMA findings, i.e. PSMA-RADS 3 A, in the prostate bed in 12/36 patients (33%), with a total median SUV_max_ of 5.1 (3.7–6.7). The total mean TBR with respect to the blood pool was 6.68 (± 3.0). Figure 1 illustrates a clinically challenging stratification of an equivocal PSMA finding assigned as PSMA-RADS 3 A in the prostate bed in a 62 years-old-patient.

We assessed the dynamics of tracer uptake in terms of SUV-metrics and TBR within both our whole cohort and the subgroup with equivocal findings (Tables 2 and 3). The assessments involving both reconstruction algorithms revealed an increase of SUV-metrics between early and delayed images by approximately 20%, which was found to be statistically significant, except TBR, on combined TOF and PSF reconstruction algorithm. Head-to-head comparisons of the SUV metrics and TBR between OSEM and combined TOF and PFS revealed a statistically significant increase in favor of combined TOF and PFS (Table 2a and b).

### Impact of the acquisition of delayed [^18^F]PSMA-1007 PET/CT imaging

A total of 12 patients (33%) were found to demonstrate equivocal [^18^F]PSMA-1007 findings (PSMA-RADS 3 A) in the prostate bed in early imaging; thus, this subgroup underwent further analyses of the SUV metric dynamics and its impact on reader confidence. The median SUV_max_ was significantly increased in delayed images by 43% (*p* < 0.001) from 3.5 (2.4–3.9) to 4.6 (2.8–6.5) on OSEM reconstruction and by 28% (*p* 0.005) from 6.6 (4.1–10.5) to 8.5 (5.0–13.8) on combined TOF and PSF reconstruction, respectively. Accordingly, TBR revealed more favorable results in delayed images (Table 3a). Since we acquired late images of only the pelvis region, a detailed, comparative analysis of biodistribution is beyond the scope of our investigation.

The favorable results with delayed [^18^F]PSMA-1007 imaging led to upgrading of reporting in 8 out of 12 patients (75%) from PSMA-RADS 3 A to PSMA-RADS 4/5. Despite a significant increase in SUV-metrics, we refrained from defining a cut-off SUV value for discriminating equivocal and clear findings, as this would contradict the main principle of the PSMA-RADS framework. Namely, this framework seeks to integrate the qualitative reading elements in a more systematic way into structured reporting in order to avoid other cut-off value associated downsides of unstructured reporting [27].

### Impact of alternative reconstruction algorithm with the combination of TOF and PSF

Compared with total cohort, the subgroup with equivocal findings (*n* = 12 patients) tended to exhibit greater dynamics with respect to SUV metrics and TBR between OSEM and combined TOF and PSF reconstruction algorithms. For instance, we observed in average a more than two-fold increase in median SUV_max_ (108%) between OSEM and combined TOF and PSF reconstructions in early imaging, namely from 3.5 (2.4–3.9) to 6.6 (4.1–10.5) (Table 3b). Compensation of PVE through the use of combined TOF and PSF reconstruction appeared to enhance the structured reporting for the small equivocal [^18^F]PSMA-1007 findings [32, 33].

**Table 2:** Total cohort overview of the dynamics of SUV-metrics and TBR in early- and delayed [^18^F]PSMA-1007 PET/CT images based upon both OSEM and combined TOF and PSF reconstruction algorithms (**a**). Head-to-head comparison of SUV metrics and TBR dynamics between reconstruction algorithms of OSEM and combination of TOF and PSF (**b**)

(a)				
Local relapse	Early phase	Late phase	Dynamic (Δ) in average	*p* value
Standard (OSEM) image reconstruction				
SUV_max_ median	5.1 (2.4–18.2)	6.8 (2.2–21.7)	+ 24.0%	< 0.001*
SUV_mean_ median	3.3 (1.5–10.6)	4.1 (1.4–12.9)	+ 17%	< 0.001*
TBR median	6.0 (2.6–14.6)	7.5 (2.8–19.7)	+ 24%	< 0.001*
Image reconstruction with combination of TOF and PSF				
SUV_max_ median	9.4 (4.1–36.4)	10.8 (4.0–40.9)	+ 20.0%	< 0.001*
SUV_mean_ median	5.1 (1.9–16.4)	6.3 (1.7–18.0)	+ 20.0%	< 0.001*
TBR median	7.9 (3.5–17.0)	9.7 (3.4–18.5)	+ 21.0%	0.205

(b)				
Local relapse	OSEM	Combination of TOF and PSF	Dynamic (Δ) in average	P value
Early phase SUV_max_ (median)	5.1 (2.4–18.2)	9.4 (4.1–36.4)	+ 94%	< 0.001*
Delayed phase SUV_max_ (median)	6.8 (2.2–21.7)	10.8 (4.0–40.9)	+ 90%	< 0.001*
Early phase SUV_mean_ (median)	3.3 (1.5–10.6)	5.1 (1.9–16.4)	+ 50%	< 0.001*
Delayed phase SUV_mean_ (median)	4.1 (1.4–12.9)	6.3 (1.7–18.0)	+ 57%	< 0.001*
Early phase TBR (median)	6.0 (2.6–14.6)	7.9 (3.5–17.0)	+ 38%	< 0.001*
Delayed phase TBR (median)	7.5 (2.8–19.7)	9.7 (3.4–18.5)	+ 35%	0.002*

*Statistically significant

**Table 3:** Overview of the SUV-metrics and TBR dynamics in the subgroup (*n* = 12 patients) with equivocal PSMA findings (PSMA-RADS 3 A) (**a**). Head-to-head comparison of SUV metrics and TBR dynamics between the OSEM and combined TOF and PSF reconstruction algorithms (**b**)

(a)				
Local relapse	Early phase	Late phase	Dynamic (Δ) in average	*P* value
Standard (OSEM) image reconstruction				
SUV_max_ median	3.5 (2.4–3.9)	4.6 (2.8–6.5)	+ 43.0%	< 0.001*
SUV_mean_ median	2.4 (1.5–3.2)	3.2 (1.9–4.7)	+ 31%	0.003*
TBR median	3.8 (2.6–5.8)	5.1 (2.8–7.7)	+ 35%	0.018*
Image reconstruction with combination of TOF and PSF				
SUV_max_ median	6.6 (4.1–10.5)	8.5 (5.0–13.8)	+ 28.0%	0.005*
SUV_mean_ median	4.0 (1.9–6.4)	4.6 (2.1–8.0)	+ 26.0%	0.001*
TBR median	6.0 (4.1–12.3)	8.2 (3.4–11.4)	+ 25.0%	0.082

(b)				
Local relapse	OSEM	Combination of TOF and PSF	Dynamic (Δ) in average	P value
Early phase SUV_max_ (median)	3.5 (2.4–3.9)	6.6 (4.1–10.5)	+ 108%	< 0.001*
Delayed phase SUV_max_ (median)	4.6 (2.8–6.5)	8.5 (5.0–13.8)	+ 86%	< 0.001*
Early phase SUV_mean_ (median)	2.4 (1.5–3.2)	4.0 (1.9–6.4)	+ 62%	< 0.001*
Delayed phase SUV_mean_ (median)	3.2 (1.9–4.7)	4.6 (2.1–8.0)	+ 62%	0.001*
Early phase TBR (median)	3.8 (2.6–5.8)	6.0 (4.1–12.3)	+ 59%	0.001*
Delayed phase TBR (median)	5.1 (2.8–7.7)	8.2 (3.4–11.4)	+ 50%	0.003*

## Discussion

Given the expected increasing use of [^18^F]PSMA-1007 as PSMA imaging of choice since the EMA-approval, there is an unmet clinical need for the enhancement of reader confidence via [^18^F]PSMA-1007 imaging in regular clinical care. [^18^F]PSMA-1007 uptake dynamics seem to depend upon the interaction of complex pharmacological factors such as PSMA receptor expression, tracer availability, its internalization and cell signaling patterns [5, 19, 34–37]. In this regard, to the best of our knowledge, this retrospective study is the first to systematically analyze the additive benefit of the aforementioned approaches in locally relapsed, castration-sensitive prostate cancer patients via [^18^F]PSMA-1007 PET/CT imaging.

This study focused on assessing the added benefit of delayed imaging and alternative reconstruction algorithms for equivocal lesions in locally relapsed prostate cancer patients by evaluating the reader confidence using a structured reporting system PSMA-RADS V2 classification. Tracer uptake dynamics was assessed in comparative manner between early and delayed images both in OSEM and combined TOF and PSF reconstruction algorithms in a cohort of patients with proven local relapse. Then we evaluated these features only in patients with equivocal findings according to retrospective reading using PSMA-RADS V2 classification.

Notably, a closer look at the tracer dynamics revealed a relatively intriguing pattern of tracer dynamics in our cohort, namely ranging from decreased uptake by 70% to increased uptake of up to 90%, which is in line with the aforementioned phenomenon [34, 35]. On the other hand, the novel reconstruction algorithm with combination of PSF and TOF leads to Gibbs artifacts overestimating SUV metrics, for instance an increase of SUV_max_ by a mean value of 23–48% compared with conventional OSEM reconstruction [33, 38–41]. Consequently, the solely use of cut-off-SUV-metrics-value for culprit lesion discrimination would not contribute to increased reader confidence in regular clinical care. Instead, the retrospective application of the structured reporting system PSMA-RADS V2 assigned 66% of cases as clear findings within early imaging, which had initially been stratified as equivocal lesions via unstructured reporting. Interestingly, the subgroup with equivocal lesions (PSMA-RADS 3 A) revealed enhanced tracer dynamics in delayed images in terms of SUV_max_ and TBR by 43% and 35%, respectively. Despite an overall enhanced tracer uptake pattern and lesion contrast, the range of these parameters was still broad, i.e. from 7% decrease up to 90% increase in SUV_max_. The structured reporting of delayed images revealed lesion upgrade to PSMA-RADS 4/5 in 75% of the patients.

Acquiring delayed images is a logistic and financial challenge for nuclear medicine facilities and somewhat liable for patients due to longer waiting times. The fact that the use of structured reporting system could help objectively identify patients who could benefit from delayed imaging would address these challenges and is promising for regular clinical care. Beyond that, the structured reporting could enhance the accurate discrimination of culprit lesions in delayed imaging. Furthermore, the pharmacokinetics of ^18^F-labelled PSMA tracers allow reasonable image quality and lesion contrast in a time window of at least 3 h [8, 37].

**Fig. 2:**
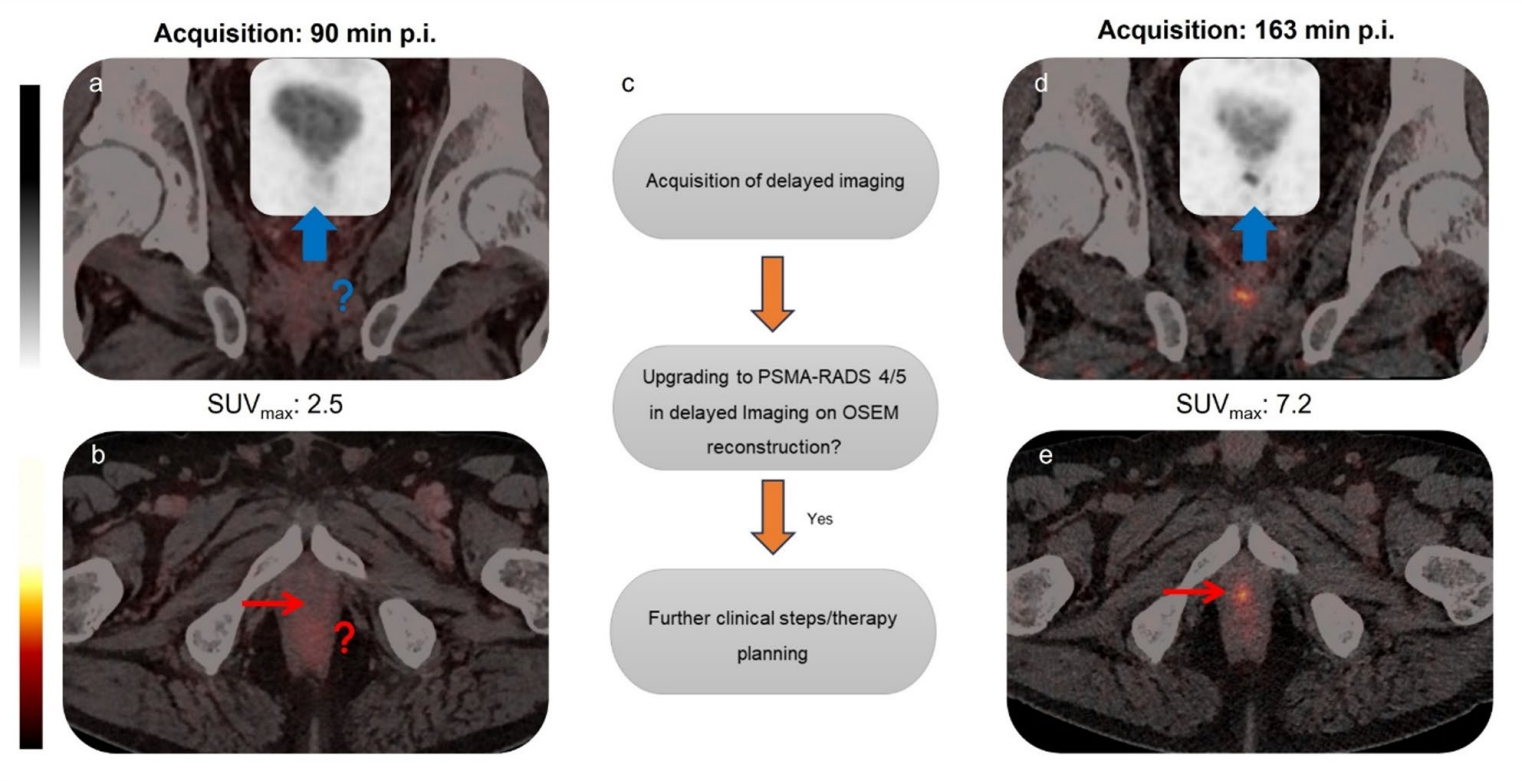
Delayed scan of a 64-years-old patient with suspected local relapse (**a** & **b**) underlined its utility by upgrading (**d** & **e**) the reporting certainty by the reader in terms of PSMA-RADS V2 (**c**)

Therefore, we evaluated also the added effect of this novel reconstruction algorithm on reader confidence. Remarkably, applied to the subgroup with PSMA-RADS 3 A findings, all lesions were upgraded to PSMA RADS 4/5 in delayed imaging which appeared to be more efficient than SUV metric dynamics (Fig. 2). In contrast to existing literature data, SUV metrics have nearly doubled in numerical value in both early and delayed images, whereas the tracer dynamics over time with both reconstruction algorithms appeared to be parallel. Furthermore, when applied to the whole cohort within the early imaging, all culprit lesions would have upgraded to PSMA-RADS 4/5 with no clinical need for further delayed imaging. This effect was also investigated by Khatri et al. for the PSMA-RADS 3 A lesions with [^18^F]DCFPyL imaging, which showed a perfect rate of 100% regarding true positivity for upgrading for the lesions, i.e.13 out of 13 upgraded lesions [14].

Its retrospective design and relatively small cohort were the main limitations our study. Since our institutional protocol foresees the conduct of delayed images in patients with equivocal findings, our cohort underlies a certain degree of selection bias. On the other hand, accurate patient pre-selection is an important integral part of personalized medicine. Furthermore, the conduct of a comparative analysis of our results with existing literature data was not possible, as similar studies for [^18^F]PSMA-1007 imaging are lacking. All eight patients upgraded on delayed imaging proceeded to salvage-directed therapy during follow-up, and findings were supported by the composite reference standard. However, the retrospective design and small cohort allowed no analysis of its clinical impact in terms of management change or survival. Prospective studies should evaluate treatment impact and outcomes. A follow-up period of at least 15 months per patient contributed to the strength of evidence, though.

**Fig. 3:**
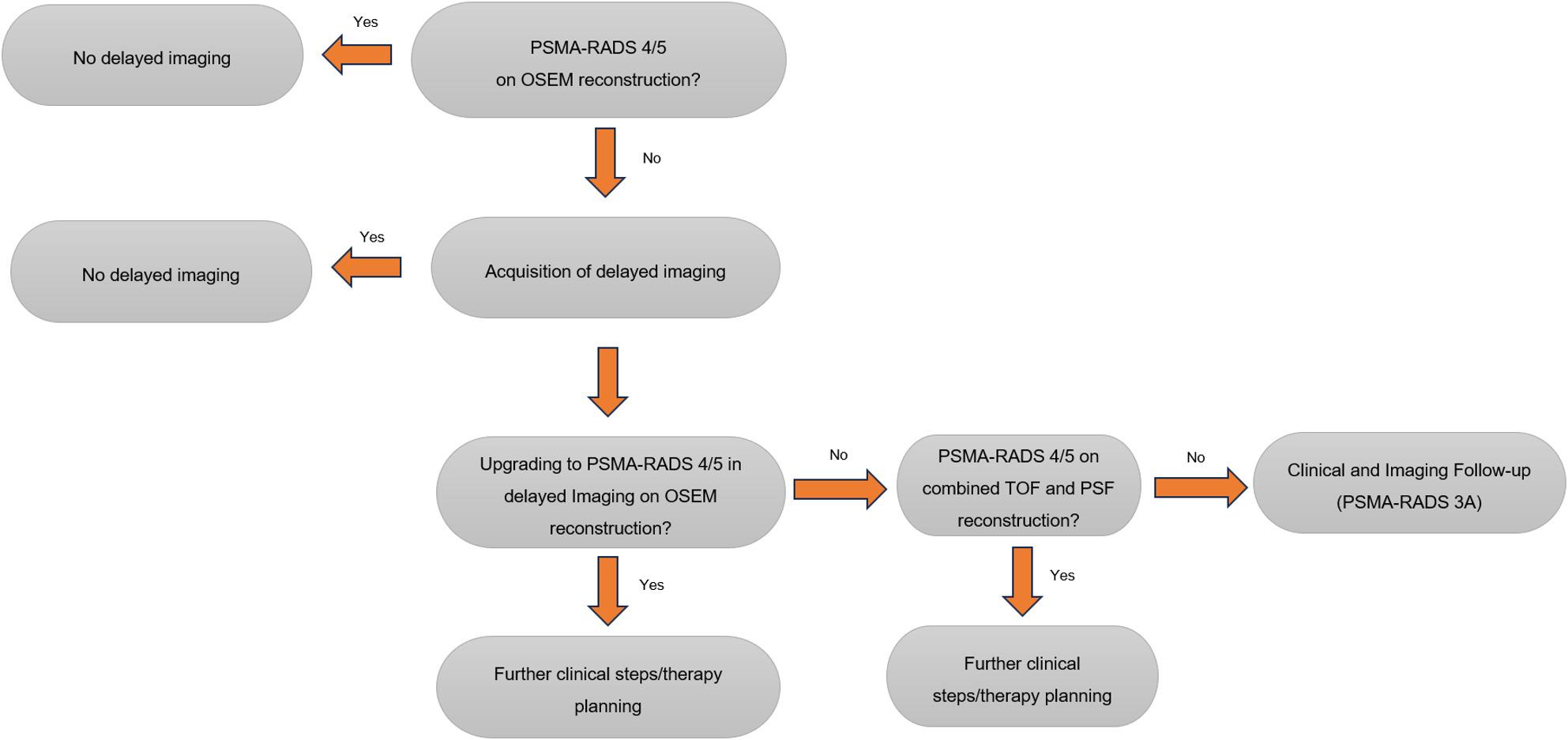
Flowchart for imaging decision-making of equivocal soft tissue lesions on [^18^F]PSMA-1007 imaging

This investigation suggests the introduction of a novel structured algorithm for reporting [¹⁸F]PSMA-1007 PET imaging. This incorporates key elements such as the additional diagnostic value of delayed imaging, the advantages of advanced reconstruction algorithms under the use of the PSMA-RADS V2 reporting system individually or in combination. This new diagnostic algorithm for PSMA-imaging, as summarized in Fig. 3, is intended to improve diagnostic confidence and support optimal patient selection for delayed imaging acquisition.

## Conclusion

This study evaluated the utility of delayed images, combined TOF and PSF reconstruction algorithm and structured reporting schema PSMA-RADS V2 to improve reader confidence and, thus, enhance the clinical decision-making process for equivocal soft tissue lesions in [^18^F]PSMA-1007 imaging. In conclusion, our results suggest the introduction of a new algorithm to enhance and streamline the imaging decision-process, which would prevent avoidable follow-up scans or further imaging modalities and spare economic resources. Yet, further large-scale studies are warranted to validate the additive effect of this algorithm in regular clinical care.

## Supplementary Information

Below is the link to the electronic supplementary material.

Supplementary Material 1

## Data Availability

The data used and/or analyzed during the current study are available from the corresponding author upon reasonable request.
