# Supplementary material for: Enhancing reader confidence through dual-time imaging and alternative reconstruction algorithms with [18F]PSMA-1007 PET/CT imaging in local relapsed castration-sensitive prostate cancer?
Source: EJNMMI Res. 2026 Mar 3;16:57. doi: 10.1186/s13550-026-01407-x (PMC13062064; doi:10.1186/s13550-026-01407-x)
Supplement: Supplementary file 1 — Supplementary Material 1 [file 13550_2026_1407_MOESM1_ESM.pdf]

Supplementary Table

**Site**	**University Hospital Düsseldorf**	
PET/CT scanner	Biograph mCT 128, Siemens	
Modality	Low dose CT	Full dose (ce)CT
CT reference (mAs)	40	190
CT peak kilovoltage (kV)	120	120
CT slice thickness (mm)	1,5	1,5
CT slice increment (mm)	1	1
PET reconstruction	OSEM algorithm	OSEM algorithm
Iterations	4	4
Subsets	8	8
Matrix	200 x 200	200 x 200
Corrections	Gaussian FWHM 2.0 mm	Gaussian FWHM 2.0 mm
